# Water–fertilizer coupling balances yield and the input-related carbon footprint in double-cropping greenhouse Hami melon

**DOI:** 10.3389/fpls.2026.1917283

**Published:** 2026-10-01

**Authors:** Zhenliang Song, Ming Hong, Quanjiu Wang, Han Guo, Fanfeng Dang, Guangning Wang, Zhaoxin Liu, Liang Ma

**Affiliations:** 1 College of Hydraulic and Civil Engineering, Xinjiang Agricultural University, Urumqi, China; 2 State Key Laboratory of Water Engineering Ecology and Environment in Arid Area, Xi’an University of Technology, Xi’an, China

**Keywords:** carbon concentration, carbon dilution effect, carbon footprint, Hami melon, structural equation model, water–fertilizer coupling

## Abstract

Water–fertilizer management is central to balancing productivity and environmental performance in greenhouse agriculture. Methods: In a spring–autumn greenhouse Hami melon system in Xinjiang, five irrigation (W1–W5) and three fertilization (F1–F3) levels were combined. Life cycle assessment quantified the input-related carbon footprint, plant carbon storage was measured, and structural equation modeling (SEM) evaluated associations among inputs, biomass, yield and carbon-footprint intensity. Results: Production inputs generated 12.77 t CO_2_-eq ha^-1^, with fertilizers (54.52%) and plastic film (23.16%) as the main sources. W4F3 achieved the highest yields (75.74 and 52.79 t ha^-1^ in spring and autumn, respectively) and plant carbon storage (up to 35.78 g C plant^-1^). In the fitted SEM, yield was strongly negatively associated with product carbon-footprint intensity (β = −1.253, P < 0.001), whereas area-based emissions were positively associated with it (β = 0.729). Under low irrigation, increasing fertilization did not consistently increase yield, while adequate water enabled fertilizer inputs to be converted more effectively into biomass and yield. Conclusion: Under the tested sandy-soil, cultivar and one-year greenhouse conditions, W4F3 (approximately 3420 m^3^ ha^-1^ irrigation and 863 kg ha^-1^ fertilizer) provided the most favorable balance between yield and product carbon-footprint intensity.

## 1 Introduction

Global agriculture is estimated to contribute a substantial share of anthropogenic greenhouse-gas (GHG) emissions, and protected cultivation has become a key sector for agricultural mitigation because it relies on intensive inputs (water, fertilizer and plastic covering) and therefore tends to exhibit higher emissions per unit area than open-field production (Tilman et al., 2002). Achieving yield stability and secure food supply while reducing environmental footprints is a central challenge for sustainable food systems (Foley et al., 2011; Willett et al., 2019).

Fertigation (integrated water and nutrient supply) can better match crop demand and has been widely adopted to increase yield and resource-use efficiency (Bar-Yosef, 1999). In China, drip fertigation has been shown to increase yield, water productivity and nitrogen partial factor productivity compared with conventional practices (Wang et al., 2019; Li et al., 2020). For melon crops, optimized water–nitrogen management can simultaneously improve yield, fruit quality and water/nitrogen use efficiency, indicating strong potential for multi-objective co-benefits (Cai et al., 2023; Zou et al., 2023; Wu et al., 2026). However, most studies focus on yield and resource efficiency, while relatively little attention has been paid to how water–fertilizer coupling affects the carbon footprint of agricultural inputs and the crop carbon sink function, especially in greenhouse fruit production (Song et al., 2026).

In greenhouse production systems, carbon footprints are typically dominated by upstream emissions from fertilizer manufacture, plastic films and irrigation electricity (Zhang et al., 2021; Li et al., 2025). Nitrogen fertilizer is often the largest contributor to embodied emissions, plastic residues have raised concerns for soil health, and pumping electricity can be a major emission source in arid regions (Brodhagen et al., 2017; Qin et al., 2024). Meanwhile, carbon sequestration through plant biomass is an important ecosystem service; water and nutrient management can regulate photosynthetic carbon assimilation and biomass accumulation, thereby influencing carbon sink potential (Lal, 2004). Integrating carbon and water-footprint perspectives can therefore provide methodological support for coordinated resource governance in water-limited regions (Shi et al., 2026). For Hami melon (Cucumis melo L.), a characteristic cash crop in Xinjiang, systematic evidence on water–fertilizer coupling effects on carbon footprints and carbon sequestration remains limited (Song et al., 2026).

This study uses the term ‘yield-associated carbon dilution’ as an interpretive description rather than as a new theory independent of fertigation optimization. Increasing irrigation and fertilization can raise emissions per unit area, but when those inputs are effectively converted into biomass and harvestable yield, the emissions allocated to each tonne of product decrease. Whether this pattern occurs therefore depends on water availability and on the efficiency with which fertilizer is translated into productive growth (Matson et al., 1998; Poore and Nemecek, 2018).

Based on a spring–autumn double-cropping greenhouse Hami melon experiment, this study aimed to: (1) quantify responses of yield, biomass, resource-use efficiency and input-related carbon-footprint intensity to irrigation and fertilization; (2) assess the trade-off between increased water–fertilizer inputs and their agronomic and environmental outputs; and (3) identify, for the tested sandy soil, cultivars and one-year greenhouse conditions, the treatment that provided the most favorable balance among yield, resource-use efficiency, plant carbon accumulation and product carbon-footprint intensity.

## 2 Materials and methods

### 2.1 Study area

The experiment was conducted in Aileketuer Village, Huayuan Township, Yizhou District, Hami City, Xinjiang, China (93°29′12.8″E, 42°46′59.7″N; 720 m a.s.l.). The region has a temperate continental arid climate, a mean annual temperature of approximately 9.8 °C, annual precipitation of about 33.8 mm and annual pan evaporation of approximately 3300 mm. Soil samples were collected from 0 to 100 cm at 20-cm intervals and the soil was classified as sandy soil (**Table 1**). **Figure 1** summarizes the greenhouse indoor meteorological record for the full experimental year: the three lines show daily maximum, minimum and mean indoor air temperature, and the bars show indoor relative humidity.

**Table 1:** Physical properties of baseline soil at the experimental site.

Soil depth (cm)	Bulk density (g cm^-3^)	Porosity (%)	Initial moisture content (%)	Initial electrical conductivity (mS/mm)	Ks (cm/day)	Soil texture
0~20	1.36	48.87	4.46	0.169	947.74	Sandy soil
20~40	1.27	52.04	15.74	0.116	1174.59	Sandy soil
40~60	1.12	57.99	22.91	0.077	919.01	Sandy soil
60~80	1.08	59.28	33.11	0.085	819.72	Sandy soil
80~100	1.24	53.37	37.58	0.133	823.80	Sandy soil

**Figure 1:**
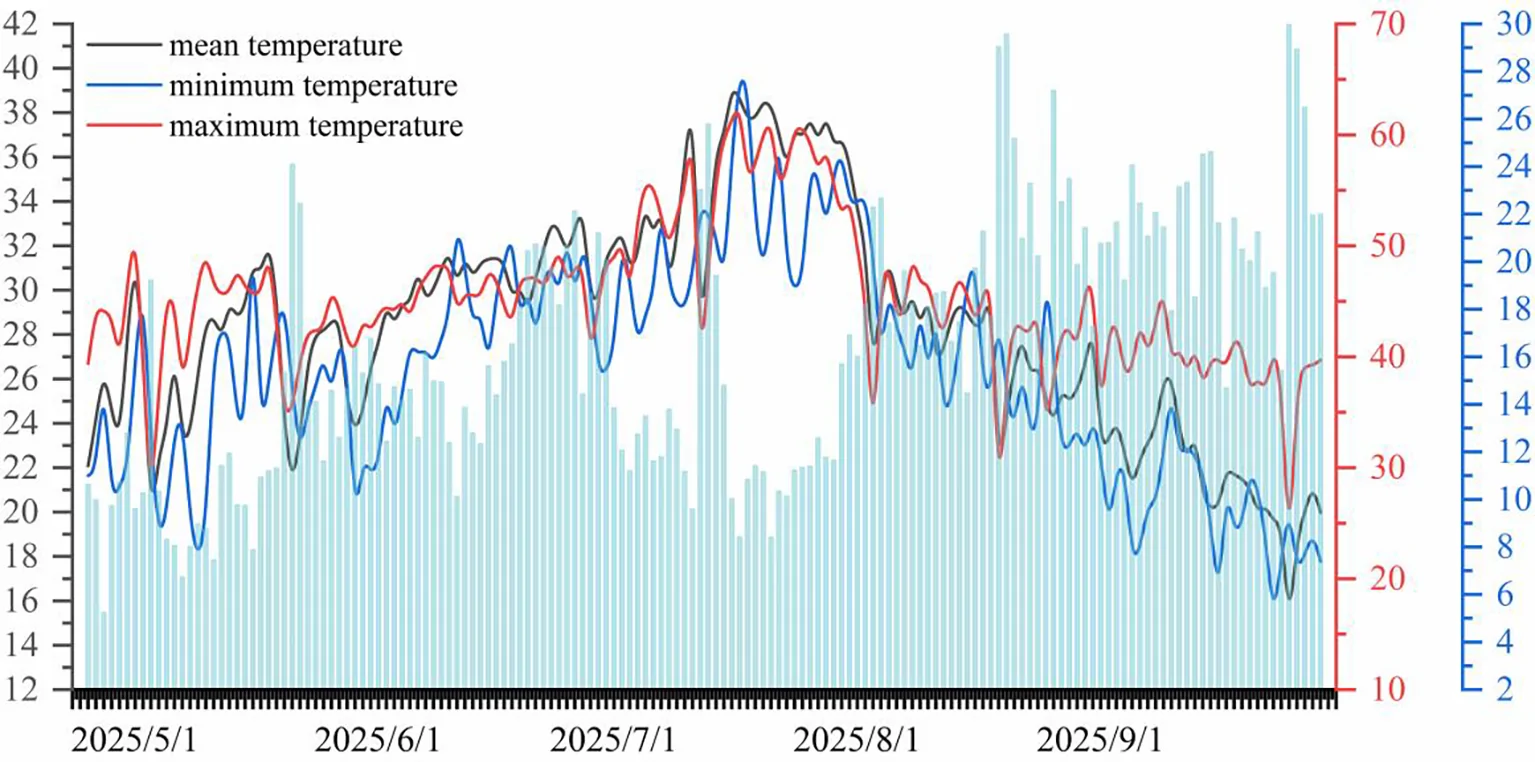
Daily maximum, minimum and mean air temperature inside the greenhouse during the full experimental year. Lines represent maximum, minimum and mean indoor temperature, and bars represent indoor relative humidity.

### 2.2 Experimental design

A spring–autumn double-cropping greenhouse Hami melon experiment was carried out from April to October 2025. Because the double-cropping schedule is constrained by seasonal temperature, radiation and rotation timing, local production typically uses combinations of cultivars with similar maturity to improve adaptation and yield stability. In this study, two early-maturing cultivars with similar growth duration, plant architecture and fertilizer demand were used: Cuican Mingzhu (spring crop) and Yugu No.2 (autumn crop), both representing locally dominant early types. Preliminary trials showed comparable responses of biomass accumulation and yield to water–fertilizer treatments between the two cultivars, supporting their use as functionally equivalent materials in this double-cropping system. Using different early-maturing cultivars in consecutive crops also reduced the risk of repeated-cropping problems: continuous cultivation of the same Hami melon cultivar in the same greenhouse can favor the accumulation of soil-borne pathogens and aggravate disease and pest pressure. The spring crop therefore used Cuican Mingzhu and the autumn crop used Yugu No.2, consistent with the local two-crop protected-production system while reducing the risk associated with consecutive cultivation of a single cultivar.

A WatchDog automatic weather station installed inside the greenhouse recorded meteorological variables, and reference evapotranspiration (*ET_0_*) was calculated using the Penman–Monteith method. Crop evapotranspiration (*ET_c_*) over the whole growth period was estimated as 3445.65 m^3^ ha^-1^ using recommended crop coefficients for Hami melon (*K_cini_* = 0.45, *K_cmid_* = 1.05, *K_cend_* = 0.80). W5 and F3 represented the local conventional high-yield irrigation quota and fertilization rate, respectively, and W5F3 was treated as the conventional control (*CK*). W1–W4 (60–90% of W5) represented progressively reduced irrigation inputs relevant to water-saving management in this arid region. F1 and F2 (80% and 90% of F3) represented moderate fertilizer-saving levels selected to avoid imposing severe nutrient deficiency that could obscure the irrigation × fertilization response. This factorial range was used to test whether fertilizer performance depended on water availability and whether inputs could be reduced without compromising productivity (**Table 2** and **3**).

**Table 2:** Irrigation and fertilization amounts during growth stages of spring-sown Hami melon.

Stage	Date	Irrigation events	Irrigation amount/(m^3^/ha)					Fertigation events	Fertilization rate/(kg/ha)		
			W1	W2	W3	W4	W5		F1	F2	F3
Seedling stage	4.25~5.4	2	135	150	180	195	225	2	108	122	135
Vining stage	5.9~5.16	4	240	300	345	390	435	4	144	162	180
Flowering stage	5.16~5.30	5	330	375	435	480	540	2	84	95	105
Fruit expansion stage	5.30~6.13	9	585	675	780	885	975	4	186	210	233
Maturity stage	6.13~6.29	15	975	1125	1320	1470	1620	6	168	189	210
Total	4.2~6.29	35	2265	2625	3060	3420	3795	18	690	777	863

**Table 3:** Irrigation and fertilization amounts during growth stages of autumn-sown Hami melon.

Stage	Date	Irrigation events	Irrigation amount/(m^3^/ha)					Fertigation events	Fertilization rate/(kg/ha)		
			W1	W2	W3	W4	W5		F1	F2	F3
Seedling stage	7.2~7.24	10	585	632	723	815	905	2	108	122	135
Vining stage	7.25~8.4	16	330	411	537	540	606	4	144	162	180
Flowering stage	8.5~8.15	10	155	380	339	459	455	2	84	95	105
Fruit expansion stage	8.16~8.31	15	728	713	798	891	1061	4	186	210	233
Maturity stage	8.31~9.16	8	437	455	621	690	710	6	168	189	210
Total	7.2~9.18	59	2234	2589	3018	3395	3735	18	690	777	863

The experiment was arranged as a two-factor irrigation × fertilization field trial with three replicates, in which irrigation and fertilization levels were combined across treatment plots. Irrigation level was assigned to main plots and fertilization level to subplots, resulting in 15 treatments (5 × 3). Each subplot measured 11 m × 4 m (44 m^2^). A protective border arrangement was used along the outermost plots. Three replicate blocks with the same layout were established within the same greenhouse, and treatments were randomly allocated within each block. The drip irrigation pipeline layout is shown in **Figure 2**.

**Figure 2:**
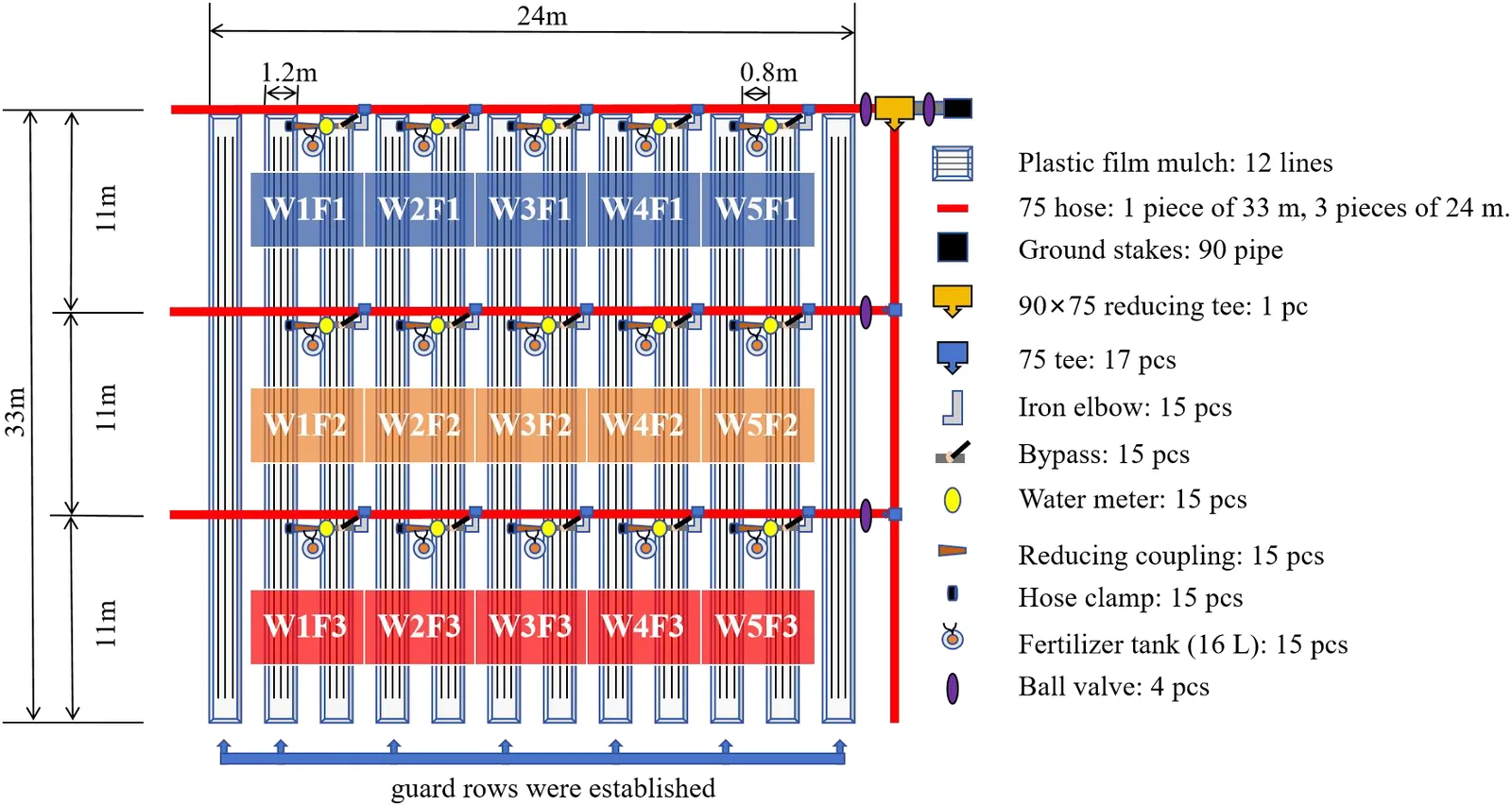
Schematic diagram of pipeline layout in the experimental area.

### 2.3 Field management

Drip irrigation was used to avoid soil crusting and organic carbon losses associated with flood irrigation, while enabling precise water supply control. Irrigation was scheduled according to crop water demand across growth stages, with particular emphasis on ensuring adequate water supply during the main vegetative accumulation period (vining and flowering/fruit set), which is a peak period for photosynthetic carbon assimilation. Seedlings were raised in a nursery. The spring crop was transplanted into the greenhouse in early April and harvested in late June; the autumn crop was sown in late June, transplanted in early July, and harvested in October. In contrast, open-field Hami melon in the region is typically single-cropped, planted in April and marketed in August. Greenhouse double cropping (early spring crop plus late autumn crop) allows off-season marketing and brings substantial economic benefits (Liu and Yang, 2024).

Hami melon was planted in two rows per bed with a film width of 1.2 m and a narrow row spacing (walkway) of 0.8 m. Given the hot, arid climate and large diurnal temperature range in Hami, unified irrigation was applied at the seedling stage to ensure emergence. Seedlings were transplanted when they reached the four-leaf stage. During the seedling hardening period, the irrigation–fertilization treatments were arranged. After transplanting, representative plants were selected using a five-point sampling method (four points on the sunny side of the film and one on the shaded side) and labeled for repeated measurements (**Figure 3**). At the vining stage, training practices (pinching, vine arrangement and trellising) were carried out. To match the staggered marketing strategy, plant spacing and pruning strategy differed between seasons: the spring crop used 30 cm plant spacing with a single main vine and one fruit per plant to accelerate fruit set and harvest, whereas the autumn crop used 60 cm plant spacing with two vines per plant to reduce seedling inputs and thus the seedling-related carbon footprint.

**Figure 3:**
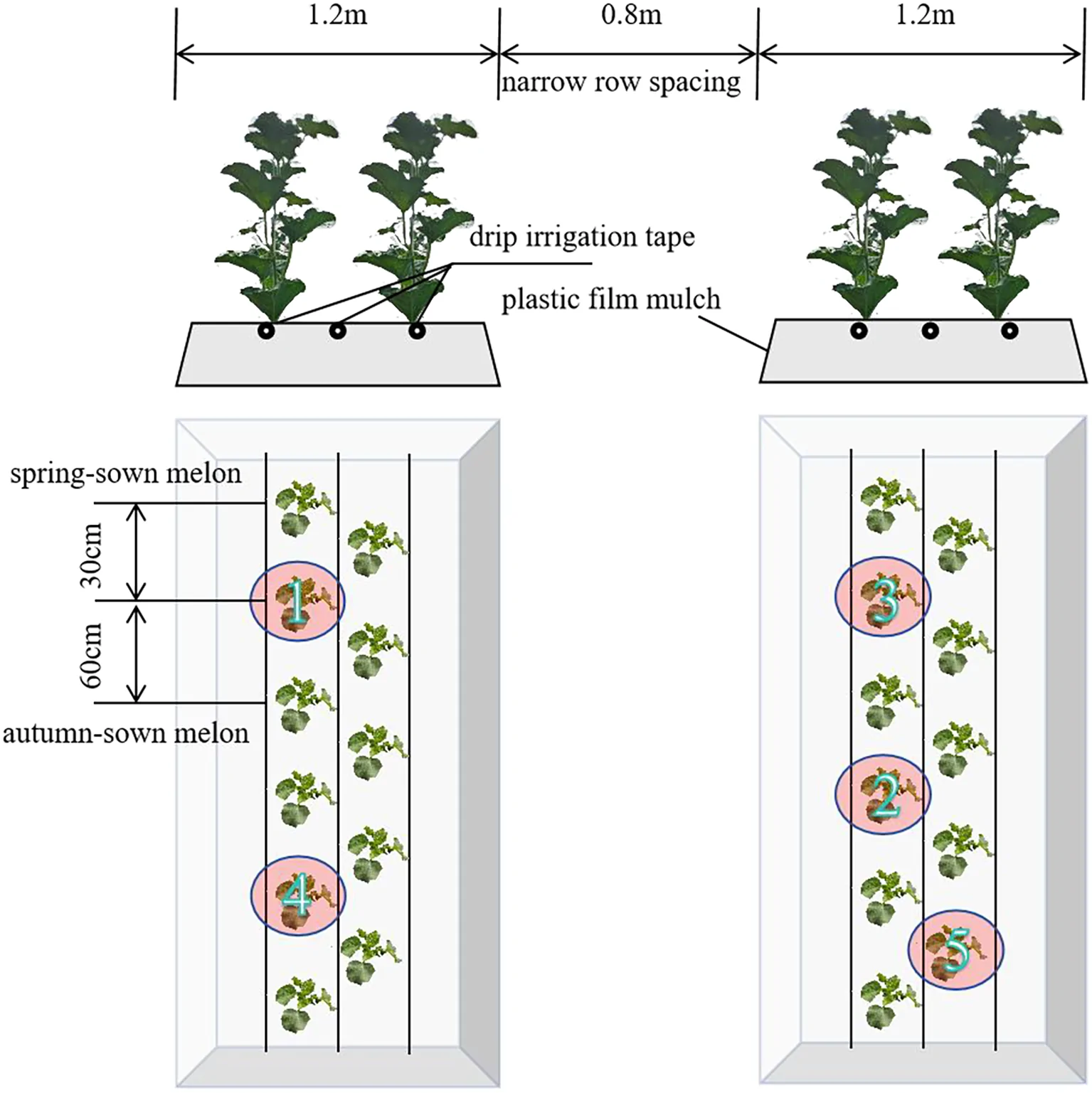
Schematic diagram of planting arrangement and the five-point sampling method.

Water-soluble fertilizers were used throughout the season: N–P_2_O_5_–K_2_O formulations of 20–20–20, 21–53–0 and 12–4–42 were applied at the corresponding growth stages. For carbon accounting, the mass of each commercial product was converted from its labelled composition to nutrient-equivalent N, P_2_O_5_ and K_2_O inputs. Fertilizer-related embodied emissions were then calculated as *CF_fert_* = ANEFN + APEFP + AKEFK + Σ(AfEFf), where AN, AP and AK are nutrient-equivalent application rates, EFN, EFP and EFK are the corresponding emission factors in **Table 4**, and AfEFf represents any separately accounted functional fertilizer. Thus, differences among fertilizer types and rates were reflected in the estimated carbon-source equivalents.

**Table 4:** Emission factors used for carbon footprint of agricultural inputs accounting.

Input item	Emission factor	Unit	Reference
Nitrogen fertilizer (nutrient basis)	8.8	kg CO_2_-eq kg^-1^	IPCC, 2019; Chen et al., 2015; Zhang et al., 2013; Ji et al., 2012
Phosphorus fertilizer (nutrient basis)	1.8	kg CO_2_-eq kg^-1^	IPCC, 2019; Chen et al., 2015; Zhang et al., 2013; Ji et al., 2012
Potassium fertilizer (nutrient basis)	0.95	kg CO_2_-eq kg^-1^	IPCC, 2019; Chen et al., 2015; Zhang et al., 2013; Ji et al., 2012
Functional water-soluble fertilizer	2	kg CO_2_-eq kg^-1^	IPCC, 2019; Chen et al., 2015; Zhang et al., 2013; Ji et al., 2012
Microbial functional fertilizer	2	kg CO_2_-eq kg^-1^	IPCC, 2019; Chen et al., 2015; Zhang et al., 2013; Ji et al., 2012
Seedlings (double cropping; half seed input in autumn)	3	kg CO_2_-eq kg^-1^	Guo et al., 2022
Pesticides (allocated across two seasons)	18.4	kg CO_2_-eq kg^-1^	Zhang et al., 2016
Plastic film (installed once; allocated across two seasons)	2.77	kg CO_2_-eq kg^-1^	Liu et al., 2025
Machinery operations (one-time; allocated across two seasons)	3.2	kg CO_2_-eq kg^-1^	SAC/TC 548, 2024; Liang et al., 2010
Electricity (allocated across two seasons)	0.61	kg CO_2_-eq kWh^-1^	Li, 2015; Wei et al., 2024

### 2.4 Measurements and calculations

#### 2.4.1 Yield and resource-use efficiency

At maturity, three fruits with similar growth status were sampled from each subplot. Yield (Y, t ha^-1^), irrigation water-use efficiency (WUE, kg m^-3^) and fertilizer partial factor productivity (PFP, kg kg^-1^) were calculated. WUE was retained because it uses directly measured irrigation and yield data. A full water footprint was not calculated because grey-water assessment requires pollutant leaching loads and background and threshold concentrations that were not measured; green water is also negligible in the covered greenhouse. This limitation is stated explicitly below.


Y=G×N/A

WUE=Y/W

PFP=Y/F

where G is mean fruit weight (t fruit^-1^), N is the number of fruits per subplot, A is subplot area (ha), Ykg is yield expressed on a mass basis (kg ha^-1^), W is total irrigation water applied (m^3^ ha^-1^), and F is total fertilizer applied (kg ha^-1^).

#### 2.4.2 Carbon footprint of agricultural inputs accounting

The system boundary was defined as transplanting to harvest. Following the principles of ISO 14040 (ISO, 2006) and relevant Chinese greenhouse-gas accounting guidance where applicable (National Technical Committee for Carbon Emission Management Standardization (SAC/TC 548), 2024), life cycle assessment quantified the embodied emissions of agricultural inputs (Einput, kg CO_2_-eq ha-1), including fertilizers, seedlings, pesticides, plastic film, machinery and irrigation electricity. The main treatment comparisons retain this input-related boundary and therefore do not present unmeasured soil N_2_O as an observed flux. To assess the possible magnitude of omitted fertilizer-induced N_2_O, a supplementary Tier 1 sensitivity estimate was made using the IPCC (2019) default dry-climate direct emission factor (EF1 = 0.005 kg N_2_O-N per kg N applied) and the 44/28 molecular-mass conversion. Based on N inputs of 147.96, 166.46 and 184.95 kg N ha^-1^ for F1, F2 and F3, respectively, direct N_2_O corresponded to approximately 0.32, 0.36 and 0.40 t CO_2_-eq ha^-1^ when expressed using GWP100 = 273. Because a default factor cannot represent irrigation-induced differences in soil moisture and no fluxes were measured, these estimates were used only to evaluate magnitude and were not merged with the treatment-level footprint or SEM. Accordingly, this study focuses on management-side (input-side) carbon-footprint optimization and does not include direct field greenhouse-gas fluxes within the main system boundary; this is a limitation of the present work.


$$E_{input}=\sum \left(A_{i}\times EF_{i}\right)$$

where Ai is the application rate of input i (kg ha^-1^ or kWh ha^-1^) and EFi is the corresponding emission factor (kg CO_2_-eq per unit input).

#### 2.4.3 Plant carbon sequestration metrics

Representative plants were sampled at the vegetative stage and at maturity. Each sampled plant was separated into roots, stems, leaves, and (at maturity) fruits. Fresh mass was recorded, samples were de-enzymed at 105 °C for 30 min, and then oven-dried at 75 °C to constant mass to obtain dry matter. Dried samples were ground and sieved, and organic carbon concentration of each organ was determined using the potassium dichromate oxidation–external heating method (Walkley and Black, 1934), following the FAO standard operating procedure for the Walkley–Black method (FAO, 2022). Organ carbon storage (g C plant^-1^) was calculated as dry matter multiplied by carbon concentration, and total plant carbon storage was the sum across organs.


$$C_{biomass}=\sum \left(DM_{j}\times C\%\right)$$

where DMj is dry matter of organ j (g plant^-1^) and C% is the corresponding organic carbon concentration.

#### 2.4.4 Area-based and product carbon footprint of agricultural inputs

The product-level input-related carbon footprint (*CF_product_*, kg CO_2_-eq t^-1^) represents the embodied emissions of the inputs included within the stated system boundary per unit yield. Area-based emissions (*CF_area_*, kg CO_2_-eq ha^-1^) were calculated as the sum of fixed inputs (*CF_fixed_*), fertilizer-related embodied emissions (CFfert) and irrigation electricity emissions (*CF_elec_*). *CF_product_* was calculated as *CF_area_* divided by yield. Accordingly, *CF_product_* is an intensity indicator mathematically dependent on both *CF_area_* and yield and is not interpreted as an independent ecosystem carbon balance.


$$CF_{area}=CF_{fixed}+CF_{fert}+CF_{elec}$$

$$CF_{product}=\frac{CF_{area}}{Y}$$

### 2.5 Statistical analysis

Data were organized in Microsoft Excel 2021, and treatment differences were evaluated in SPSS using the LSD test at P < 0.05. For SEM, a dimensionless composite water–fertilizer input index (WFI) was constructed for descriptive integration of the two treatment factors: Wn = Wi/WCK, Fn = Fi/FCK, and WFI = √(Wn × Fn), where Wi and Fi are the irrigation and fertilization inputs for treatment i and WCK and FCK are those of W5 and F3; thus W5F3 has WFI = 1. WFI represents relative combined input intensity and is not an independently measured physiological or ecological process. SEMs evaluated associations among WFI, biomass, yield and carbon-footprint intensity. Model fit was assessed using the comparative fit index (CFI) and root mean square error of approximation (RMSEA); CFI > 0.90 and RMSEA < 0.08 were treated as acceptable, while CFI > 0.95 and RMSEA < 0.05 indicated good fit (Hu and Bentler, 1999).

## 3 Results

### 3.1 Composition of carbon footprint inputs

In the spring–autumn double-cropping system, embodied production inputs generated 12.77 t CO_2_-eq ha^-1^. Fertilizers were the largest source (54.52%), followed by plastic films (23.16%) and irrigation electricity (15.96%) (**Figure 4**). Consistent with previous agricultural LCA studies, the dominance of fertilizer-related emissions is treated here as a contextual hotspot rather than as a novel finding. The analytical contribution is the treatment-level assessment of how input-related emissions interact with biomass and yield to determine product carbon-footprint intensity.

**Figure 4:**
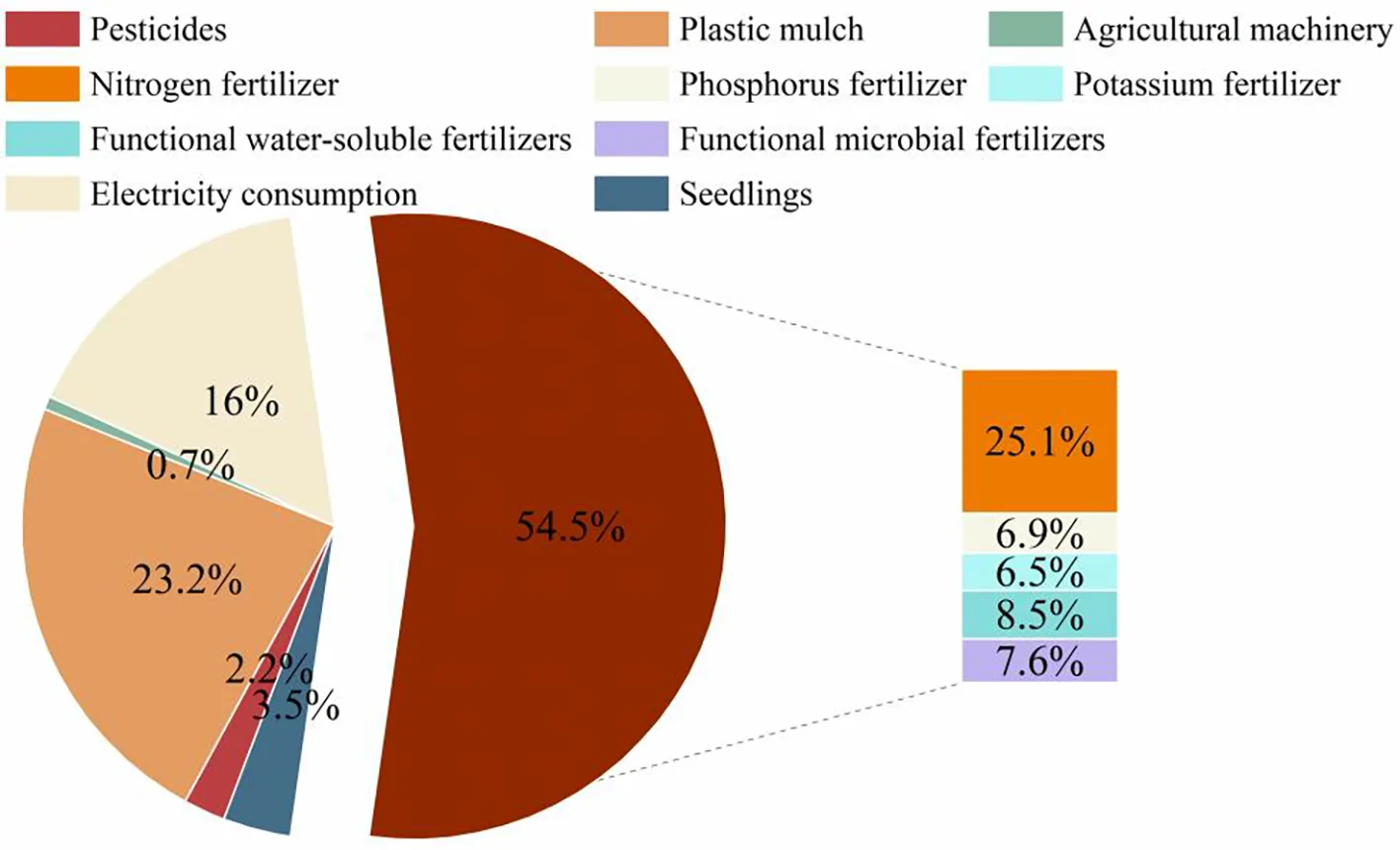
Contribution of different inputs to the carbon footprint of agricultural inputs in the spring–autumn double-cropping greenhouse Hami melon system.

### 3.2 Effects of irrigation–fertilization coupling on yield and resource-use efficiency

Irrigation, fertilization and their interaction had highly significant effects on yield, water use efficiency (WUE) and fertilizer partial factor productivity (PFP) in both seasons (P < 0.01; **Table 5**). Yield was maximized by W4F3 in both seasons (75.74 t ha^-1^ in spring and 52.79 t ha^-1^ in autumn), representing yield increases of 9.71% and 6.24% relative to the conventional control (CK), respectively. Under low irrigation (W1), increasing fertilization (F1 to F3) did not continuously increase yield; in the spring crop, W1F3 (50.18 t ha^-1^) was significantly lower than W1F2 (59.62 t ha^-1^), indicating that sufficient water supply is a prerequisite for fertilizer benefits.

**Table 5:** Effects of irrigation and fertilization treatments on yield and resource-use efficiency of Hami melon.

Treatment	Spring-sown melon			Autumn-sown melon		
	Yield (t ha^-1^)	WUE (kg m^-3^)	PFP (kg kg^-1^)	Yield (t ha^-1^)	WUE (kg m^-3^)	PFP (kg kg^-1^)
W1F1	40.82 ± 1.92h	18.02 ± 0.86de	59.16 ± 2.82f	28.40 ± 0.88f	12.72 ± 0.39de	41.17 ± 1.27d
W1F2	59.62 ± 2.17de	26.31 ± 0.96a	76.73 ± 2.86cde	37.13 ± 2.18de	16.62 ± 1.05b	47.77 ± 3.01cd
W1F3	50.18 ± 0.62g	22.15 ± 0.26bc	58.15 ± 0.78f	28.54 ± 0.97f	12.78 ± 0.46de	33.06 ± 1.20e
W2F1	50.52 ± 2.19g	19.26 ± 0.83d	73.22 ± 3.34de	29.93 ± 3.11f	11.56 ± 1.23ef	43.39 ± 4.60d
W2F2	63.53 ± 1.79cde	24.20 ± 0.68ab	81.76 ± 2.30abcd	48.73 ± 0.82ab	18.82 ± 0.33a	62.71 ± 1.10a
W2F3	58.76 ± 3.68f	22.39 ± 1.38b	68.09 ± 4.29e	45.28 ± 0.92bc	17.49 ± 0.36ab	52.47 ± 1.08bc
W3F1	60.68 ± 2.13ef	19.83 ± 0.66cd	87.94 ± 3.23ab	32.02 ± 2.50ef	10.61 ± 0.83fg	46.41 ± 3.63cd
W3F2	66.92 ± 2.03cde	21.87 ± 0.63bc	86.12 ± 2.64abc	48.75 ± 0.70ab	16.15 ± 0.22b	62.73 ± 0.86a
W3F3	72.28 ± 0.69ab	23.62 ± 0.23b	83.75 ± 0.80abc	52.27 ± 0.19a	17.32 ± 0.06ab	60.56 ± 0.21a
W4F1	61.52 ± 4.28def	17.99 ± 1.26de	89.15 ± 6.53ab	40.23 ± 2.92cd	11.85 ± 0.86ef	58.30 ± 4.24ab
W4F2	66.09 ± 0.67cd	19.33 ± 0.19d	85.06 ± 0.82abc	48.33 ± 0.84ab	14.24 ± 0.26cd	62.19 ± 1.12ab
W4F3	75.74 ± 0.24a	22.15 ± 0.06bc	87.77 ± 0.27ab	52.79 ± 0.74a	15.55 ± 0.23bc	61.18 ± 0.89a
W5F1	62.83 ± 1.13de	16.56 ± 0.27e	91.06 ± 1.70a	35.89 ± 3.48de	9.61 ± 0.95g	52.03 ± 5.16bc
W5F2	66.05 ± 1.59cd	17.40 ± 0.37de	85.00 ± 2.03abc	48.50 ± 0.67ab	12.99 ± 0.19de	62.43 ± 0.89a
CK	69.02 ± 3.10bc	18.19 ± 0.76de	79.98 ± 3.73bcd	49.69 ± 0.48ab	13.31 ± 0.13de	57.59 ± 0.58ab

Different letters within the same column indicate significant differences among treatments at P < 0.05. W1–W5 indicate increasing irrigation levels; F1–F3 indicate increasing fertilization levels; CK indicates conventional farmer practice.

WUE maxima differed between seasons: W1F2 had the highest WUE in spring (26.31 kg m^-3^), whereas W2F2 was highest in autumn (18.82 kg m^-3^). However, treatments with the highest WUE did not coincide with the highest yields, indicating a typical trade-off between maximizing resource efficiency and maximizing yield. In contrast, W4F3 achieved high yield with intermediate WUE (22.15 kg m^-3^ in spring; 15.55 kg m^-3^ in autumn). These results distinguish the multi-objective optima: W4F3 was the best-performing treatment for yield maximization and low product carbon-footprint intensity, whereas W1F2 in spring and W2F2 in autumn were the respective WUE optima.

PFP was primarily regulated by fertilization level and generally decreased as fertilization increased (F1 > F2 > F3), consistent with diminishing returns. Notably, under low irrigation (W1), moderate fertilization (F2) yielded higher PFP than F1 (e.g., 76.73 vs 59.16 kg kg^-1^ in spring), suggesting that a moderate increase in fertilization may temporarily improve fertilizer productivity when water is limiting. However, excessive fertilization (F3) sharply reduced PFP. Under medium to high irrigation (W3–W5), the negative effect of increased fertilization on PFP was attenuated, further confirming that adequate water supply underpins efficient fertilizer conversion.

### 3.3 Plant carbon accumulation under irrigation–fertilization coupling

#### 3.3.1 Dry matter accumulation

Water and fertilizer management significantly affected dry matter accumulation in various organs and the net accumulation over the growth period (**Table 6**). For comparative purposes, we defined the difference in dry matter between the vegetative and maturity stages as the “Cumulative Net Carbon Sequestration,” reflecting the net increase in carbon assimilation products during the late growth period.

**Table 6:** Dry matter accumulation (roots, stems and leaves combined) at the vegetative stage and maturity under different treatments.

Treatment	Dry matter content of spring-sown melon			Dry matter content of autumn-sown melon		
	Vegetative stage (g)	Maturity stage (g)	Total dry matter accumulation (g)	Vegetative stage (g)	Maturity stage (g)	Total dry matter accumulation (g)
W1F1	19.60 ± 0.57j	36.10 ± 1.04g	16.50 ± 0.48i	17.00 ± 0.49g	36.10 ± 1.04g	19.10 ± 0.55cd
W1F2	31.07 ± 0.90g	36.00 ± 1.04g	4.93 ± 0.14m	28.66 ± 0.83f	38.20 ± 1.10g	9.54 ± 0.27h
W1F3	22.96 ± 0.66i	30.40 ± 0.88h	7.44 ± 0.21l	26.00 ± 0.75f	36.50 ± 1.05g	10.50 ± 0.30gh
W2F1	26.03 ± 0.75hi	44.50 ± 1.28f	18.47 ± 0.54h	25.80 ± 0.74f	38.40 ± 1.11g	12.60 ± 0.36f
W2F2	45.01 ± 1.30bc	56.10 ± 1.62e	11.09 ± 0.32k	41.00 ± 1.18cd	53.70 ± 1.55e	12.70 ± 0.37f
W2F3	42.59 ± 1.23cde	62.00 ± 1.79cd	19.41 ± 0.56gh	35.00 ± 1.01e	46.50 ± 1.34f	11.50 ± 0.33fg
W3F1	27.11 ± 0.78h	59.20 ± 1.71de	32.09 ± 0.92a	39.30 ± 1.13cd	58.10 ± 1.68de	18.80 ± 0.54cde
W3F2	46.60 ± 1.35ab	59.70 ± 1.72cde	13.10 ± 0.38j	41.60 ± 1.20c	59.20 ± 1.71cd	17.60 ± 0.51de
W3F3	47.47 ± 1.37ab	60.50 ± 1.75cde	13.03 ± 0.38j	49.60 ± 1.43b	69.60 ± 2.01b	20.00 ± 0.58bc
W4F1	35.78 ± 1.03f	59.20 ± 1.71de	23.42 ± 0.68cd	37.80 ± 1.09de	61.00 ± 1.76cd	23.20 ± 0.67a
W4F2	44.18 ± 1.28bcd	68.20 ± 1.97ab	24.02 ± 0.69bc	48.70 ± 1.41b	71.80 ± 2.07ab	23.10 ± 0.67a
W4F3	48.68 ± 1.41a	70.90 ± 2.05a	22.22 ± 0.64de	56.50 ± 1.63a	75.30 ± 2.17a	18.80 ± 0.54cde
W5F1	39.43 ± 1.14e	65.00 ± 1.88bc	25.57 ± 0.74b	38.60 ± 1.11cd	62.10 ± 1.79cd	23.50 ± 0.68a
W5F2	41.42 ± 1.20de	62.80 ± 1.81cd	21.38 ± 0.62ef	41.20 ± 1.19cd	62.00 ± 1.79cd	20.80 ± 0.60b
CK	49.68 ± 1.43a	70.00 ± 2.02ab	20.32 ± 0.59fg	47.10 ± 1.36b	64.40 ± 1.86c	17.30 ± 0.50e

The lowercase letters represent homogeneous subsets obtained by the LSD‑Duncan post‑hoc multiple comparisons following one‑way analysis of variance (ANOVA). Treatments sharing any common lowercase letter are not significantly different at P<0.05. Error bars denote standard errors of the mean.

Inhibitory effect of low water and high fertilizer: Under water-limited conditions (W1, W2), increasing the fertilizer application rate not only failed to consistently promote dry matter accumulation but, in most treatments, significantly inhibited the cumulative net carbon sequestration. Taking the spring planting as an example, under the W1 treatment, the cumulative net carbon sequestration decreased sharply with increasing fertilizer rate, from 16.50 g in F1 to 4.93 g in F2 and 7.44 g in F3. Under the W2 treatment, the cumulative net carbon sequestration in F2 (11.09 g) was also significantly lower than that in F1 (18.47 g). This indicates that under water stress, excessive fertilization exacerbates the physiological burden on plants, inhibiting the transport and storage of photosynthetic products to reproductive organs. Synergistic effect of medium to high water: Under sufficient water conditions (W3–W5), the inhibitory effect of increased fertilizer on the cumulative net carbon sequestration largely disappeared, and in some cases, even showed a promoting effect. For instance, in the spring planting under the W4 treatment, the cumulative net carbon sequestration for F1–F3 remained stable between 22.22 and 24.02 g. In the autumn planting under the W4 treatment, the cumulative net carbon sequestration for F1 and F2 both exceeded 23 g, and while F3 was slightly lower, it remained higher than most low-water treatments. This confirms that water availability is a key factor limiting fertilizer synergies and sustained carbon fixation.

#### 3.3.2 Comparison of fresh and dry biomass

**Figure 5** compares fresh mass and dry mass of plants across treatments at the vegetative stage and maturity. The results indicate that irrigation and fertilization conditions not only affected biomass magnitude, but also altered tissue water status. Overall, under low irrigation (W1 and W2), increased fertilization provided only limited gains in total dry mass at maturity (in the spring crop under W1, maturity dry mass ranged only from ~30 to 36 g), and some high-fertilizer treatments (e.g., W1F3) even had lower dry mass than low-fertilizer treatments (W1F1), consistent with a pattern of “early luxuriant growth but late-stage inhibition”.

**Figure 5:**
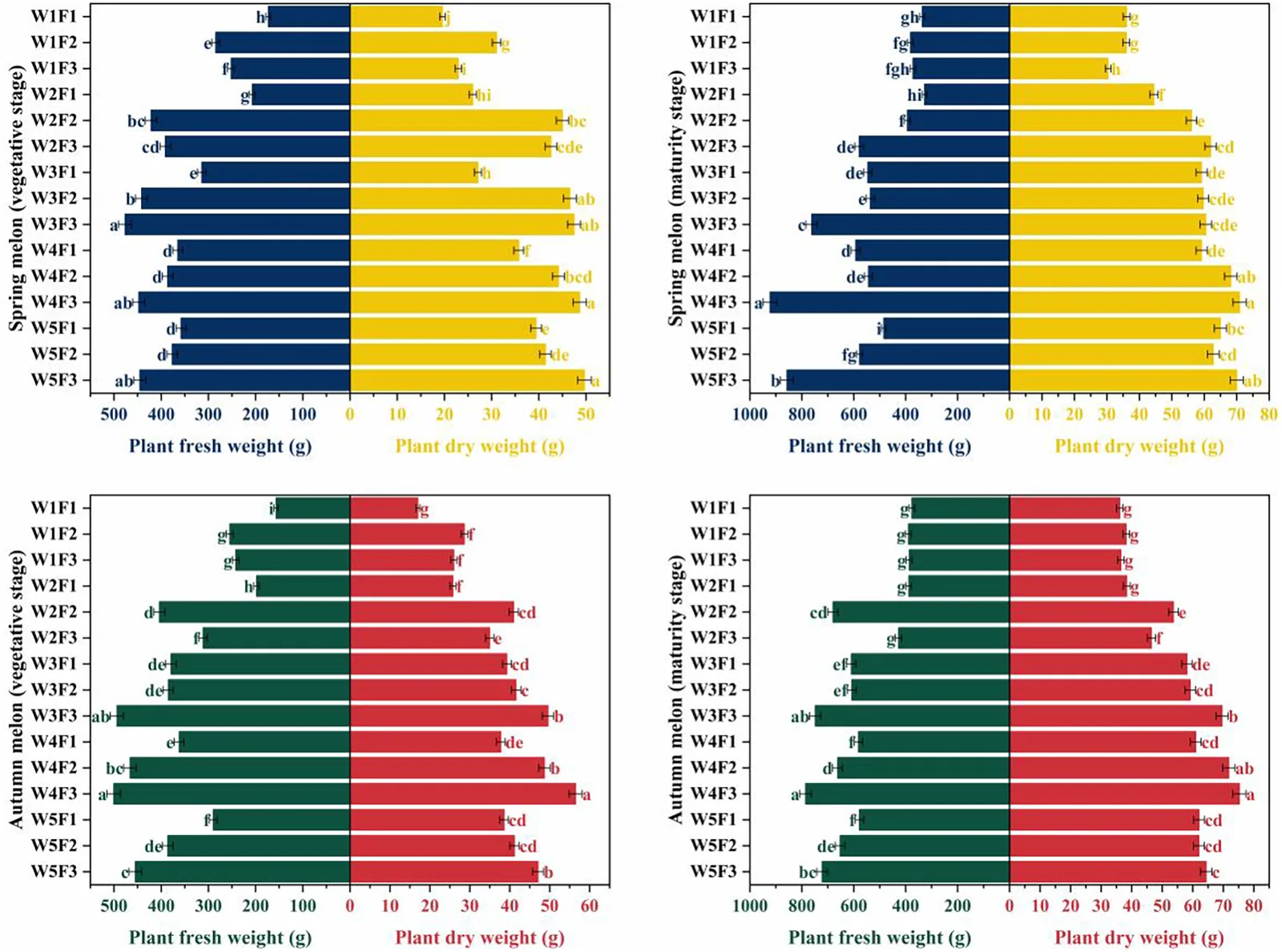
Comparison of fresh and dry biomass of Hami melon plants under different treatments across two seasons. The lowercase letters represent homogeneous subsets obtained by the LSD‑Duncan post‑hoc multiple comparisons following one‑way analysis of variance (ANOVA). Treatments sharing any common lowercase letter are not significantly different at P<0.05. Error bars denote standard errors of the mean.

The reduction in fresh mass under low irrigation was larger than the reduction in dry mass, indicating a degree of “carbon concentration” (i.e., a higher proportion of dry matter per unit fresh mass). In contrast, under medium-to-high irrigation, tissue water content was higher but total dry matter increased substantially, suggesting a carbon accumulation pathway dominated by biomass expansion. Consistent with the plant carbon storage results, carbon concentration alone did not guarantee higher total carbon storage; final carbon sequestration depended on total dry matter accumulation.

#### 3.3.3 Plant carbon storage

Plant carbon storage (**Figure 6**) further revealed carbon allocation strategies under different water–fertilizer regimes.Under low irrigation, plants exhibited a “carbon concentration” pattern: organ carbon concentration could remain relatively high, but total carbon storage remained low because biomass accumulation was constrained. This stress-induced defensive strategy sacrifices growth and yield and therefore contributes little to the overall carbon sink function.

**Figure 6:**
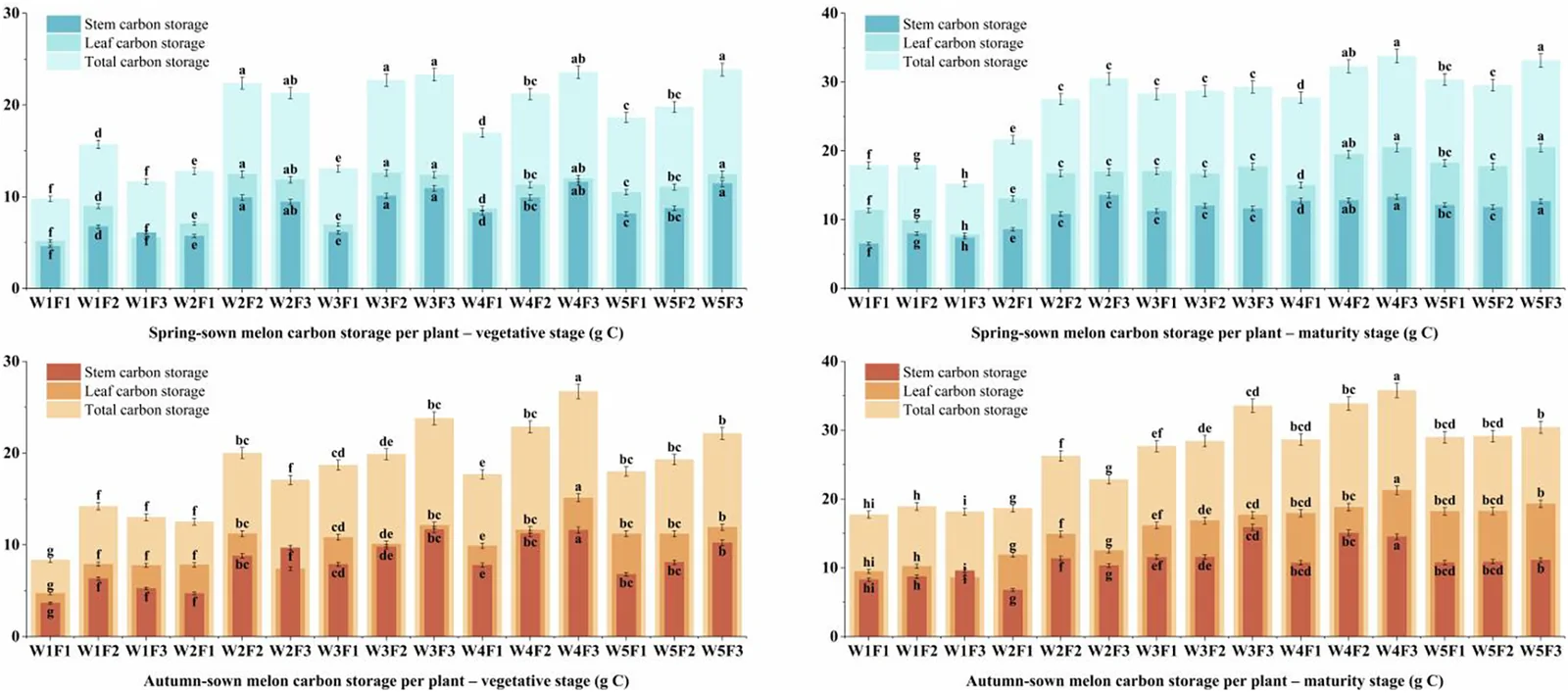
Comparison of plant carbon storage under different treatments across two seasons. The lowercase letters represent homogeneous subsets obtained by the LSD‑Duncan post‑hoc multiple comparisons following one‑way analysis of variance (ANOVA). Treatments sharing any common lowercase letter are not significantly different at P<0.05. Error bars denote standard errors of the mean.

The highest plant carbon storage in both seasons occurred under W4F3. Adequate irrigation supported high photosynthetic rates and biomass production, and high fertilization was efficiently utilized, resulting in the maximum total carbon storage at maturity (33.78 g C plant^-1^ in spring; 35.78 g C plant^-1^ in autumn). The corresponding carbon sequestration potential reached 1126–1192 kg C ha^-1^, indicating a shift from “defensive” carbon concentration under stress to “productive” carbon accumulation under optimized management.

### 3.4 Fruit carbon accumulation

Fruit carbon storage generally increased as irrigation and fertilization improved. Fruit carbon storage ranged from 79.81 to 181.64 g C fruit^-1^ in the spring crop and from 59.42 to 137.83 g C fruit^-1^ in the autumn crop. The highest fruit carbon storage in both seasons occurred under W4F3 (181.64 g C fruit^-1^ in spring; 137.83 g C fruit^-1^ in autumn), indicating that high irrigation and high fertilization not only enhanced yield but also strengthened carbon allocation to fruits. This yield increase coupled with enhanced carbon export to fruits provides a biological basis for reducing carbon footprint intensity, because higher product output is obtained per unit emission. Fruit carbon concentration remained relatively stable (approximately 47–49%) across treatments (**Figure 7**).

**Figure 7:**
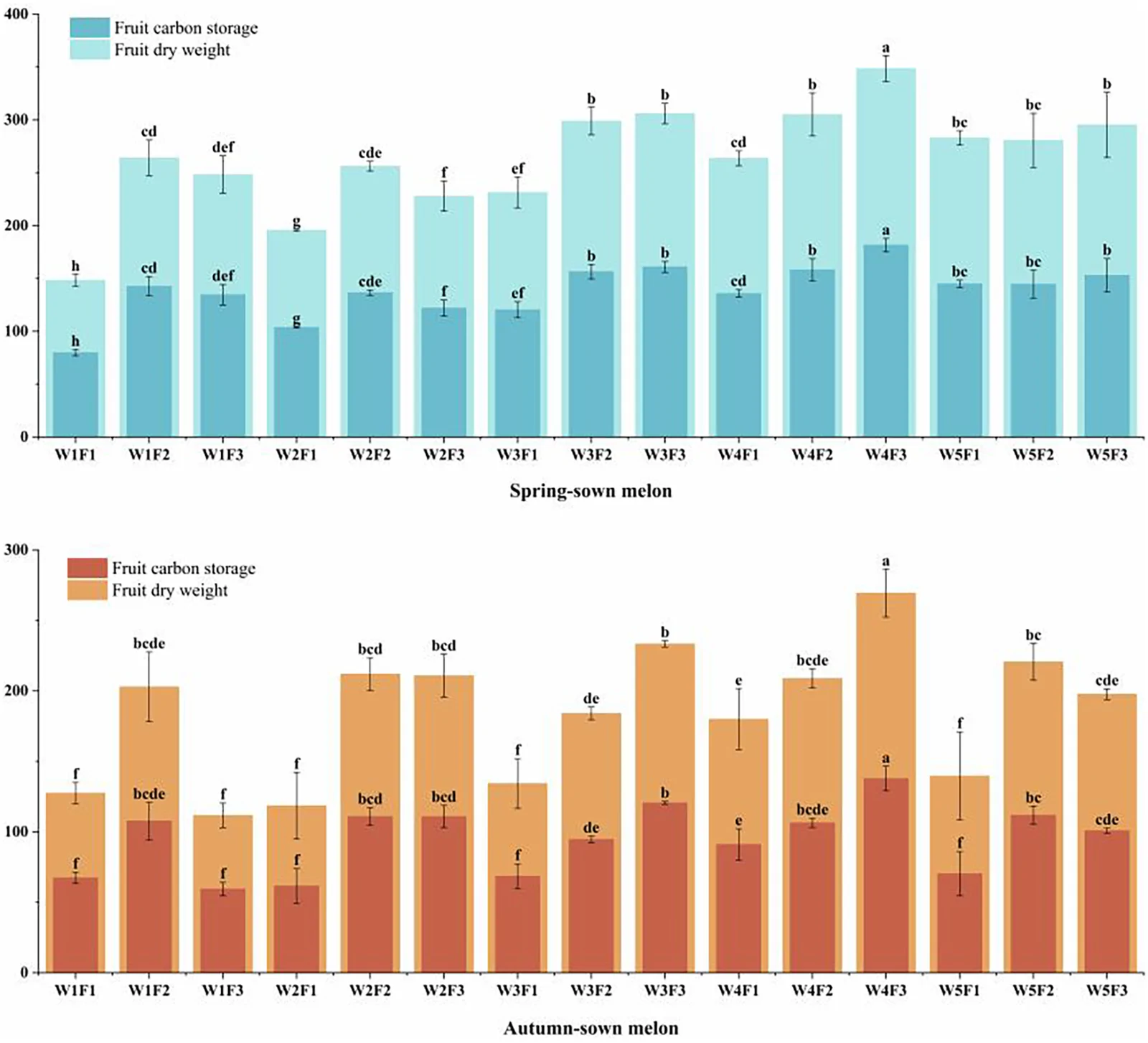
Comparison of fruit carbon storage under different treatments across two seasons. The lowercase letters represent homogeneous subsets obtained by the LSD‑Duncan post‑hoc multiple comparisons following one‑way analysis of variance (ANOVA). Treatments sharing any common lowercase letter are not significantly different at P<0.05. Error bars denote standard errors of the mean.

### 3.5 Relationships between yield and carbon footprints under different treatments

**Figure 8** shows a consistent inverse yield–*CF_product_* pattern across the spring and autumn crops rather than distinct treatment clusters. Although absolute yields differed between seasons, treatments with higher yield generally had lower product carbon-footprint intensity. W4F3 combined the highest yield with the lowest or near-lowest *CF_product_* in both seasons, while W4F2 provided a comparatively stable resource-saving option. The figure is therefore interpreted as evidence of cross-season directional consistency, not as a clustering analysis.

**Figure 8:**
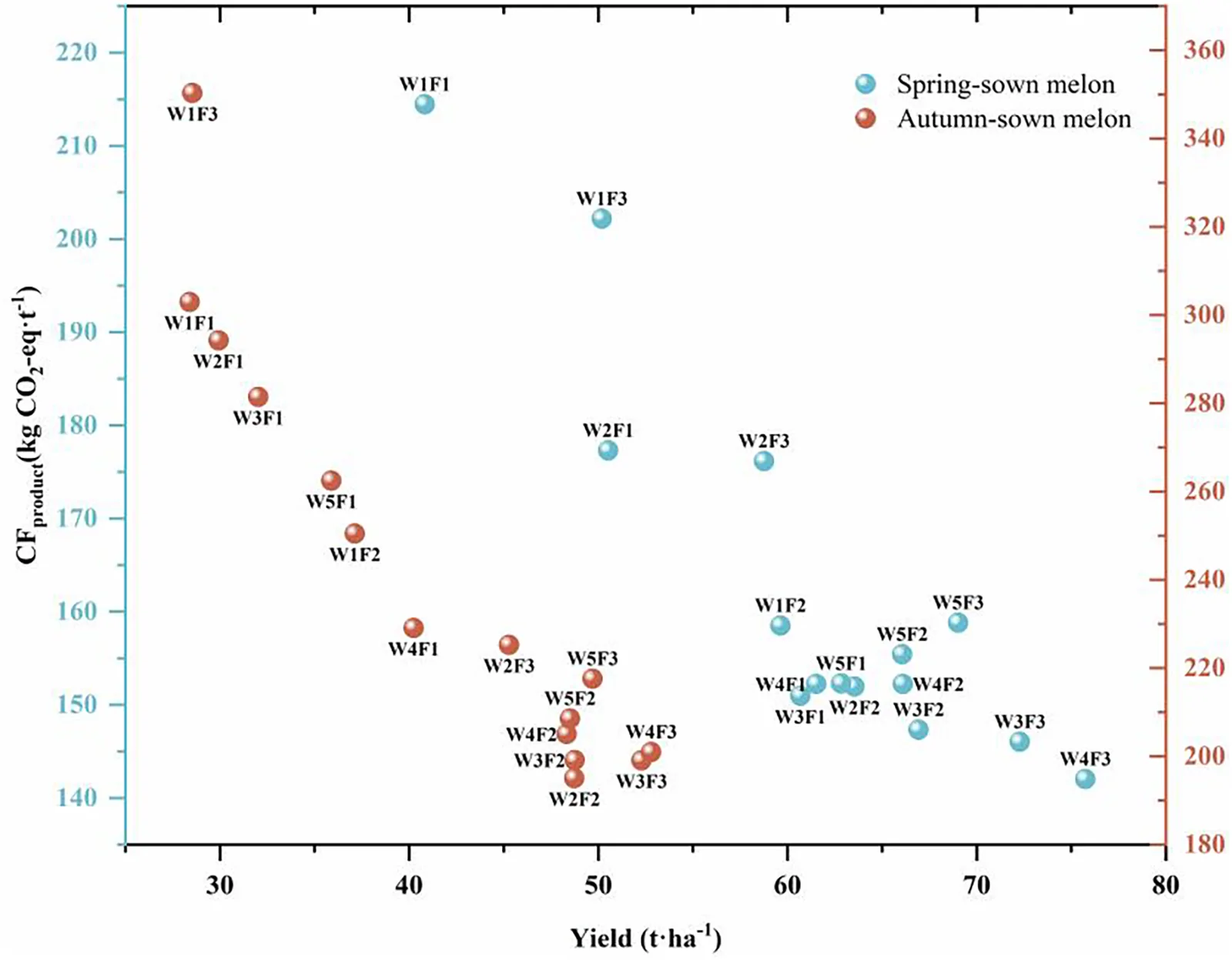
Relationship between yield and the product-level input-related carbon footprint (*CF_product_*) across treatments in the spring and autumn crops. The figure is used to compare the direction and consistency of the yield–*CF_product_* trend between seasons; no clustering analysis is inferred.

The interaction between irrigation and fertilization was clear. Under low irrigation (W1), increasing fertilization (F1 to F3) markedly increased *CF_product_*; in the autumn crop, *CF_product_* reached 350.32 kg CO_2_-eq t^-1^ under W1F3, supporting the conclusion that adding fertilizer under water limitation suppresses carbon accumulation while increasing emissions. Under medium-to-high irrigation (W3–W5), increasing fertilization maintained or reduced *CF_product_*, indicating that adequate water supply is a prerequisite for realizing yield gains and carbon benefits from fertilization.

By contrast, the area-based carbon footprint (*CF_area_*) increased monotonically with higher water and fertilizer inputs (8753.95–10962.13 kg CO_2_-eq ha^-1^ in spring; 8604.15–10812.33 kg CO_2_-eq ha^-1^ in autumn), confirming that intensification directly increases emissions per unit area. High-yield treatments nevertheless achieved lower *CF_product_* because higher yield effectively diluted the carbon cost per unit product. The complete treatment-level *CF_area_* values are provided in **Supplementary Table S1**.

### 3.6 Structural equation modeling of water–fertilizer coupling, yield and carbon footprints

The pooled SEM (**Figure 9**; CFI = 0.972, RMSEA = 0.041) included WFI, vegetative- and maturity-stage biomass, yield, plant carbon storage, *CF_area_* and *CF_product_*. Because WFI is calculated from irrigation and fertilizer inputs and *CF_area_* includes fertilizer and irrigation-electricity emissions, a positive WFI–*CF_area_* association is partly expected from shared mathematical inputs. This path is therefore used descriptively to represent increasing input intensity, not as evidence of an independent causal mechanism. The more informative paths concern whether increasing combined inputs were associated with biomass and yield formation.

**Figure 9:**
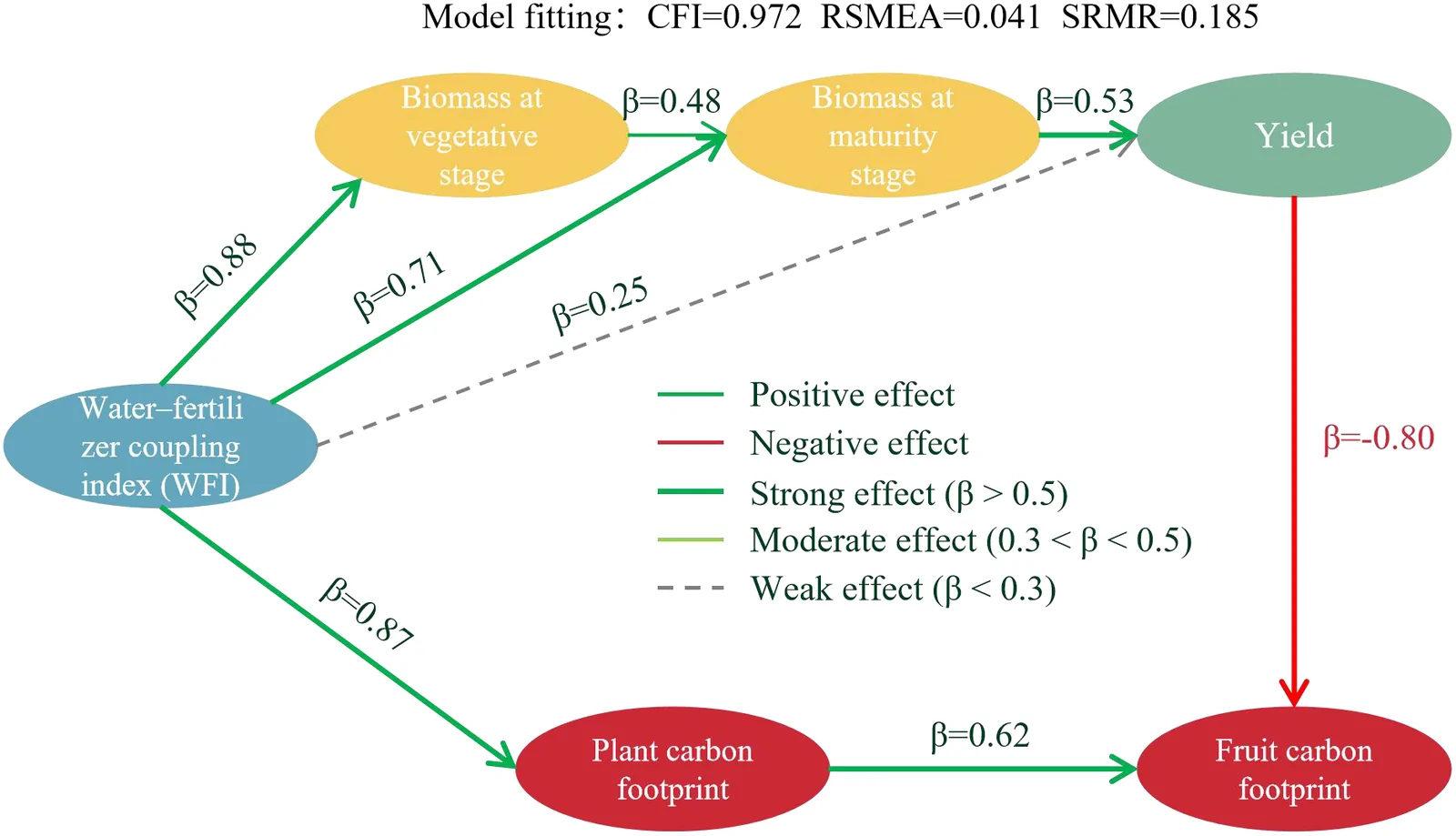
Pooled structural equation model showing statistical pathways among the composite water–fertilizer input index (WFI), biomass, yield and the input-related carbon-footprint indicators. WFI represents relative combined input intensity and is not an independently measured process.

Yield formation pathway: WFI promoted yield through both a direct effect (β = 0.485) and an indirect effect via biomass accumulation (WFI → vegetative biomass, β = 0.457; vegetative biomass → maturity biomass, β = 0.413). This pathway represents the physiological basis for yield improvement under water–fertilizer coupling. The same SEM structure was also fitted to pooled two-season data. Results were consistent with the seasonal models (CFI = 0.972, RMSEA = 0.041), confirming that the pathway patterns were robust across seasons.

Emission pathway: WFI had a strong positive direct effect on *CF_area_* (β = 0.950), indicating that intensified irrigation and fertilization directly increased total emissions per unit area.

Within the fitted pooled SEM, *CF_product_* was positively associated with *CF_area_* (β = 0.729) and negatively associated with yield (β = −1.253). Because *CF_product_* is defined as *CF_area_* divided by yield, these coefficients partly reflect that deterministic relationship and should not be interpreted as independent causal effect sizes. They indicate that, within the observed treatment range, yield-associated variation in product intensity was larger in magnitude than the positive association with area-based emissions.

Carbon sink pathway: WFI strongly increased plant carbon storage (β = 0.862), but plant carbon storage had no significant direct effect on *CF_product_* (P > 0.05). This suggests that plant carbon sequestration does not directly reduce *CF_product_* within the defined system boundary; rather, reductions are mainly achieved indirectly through yield dilution.

The direct WFI–*CF_product_* path was not significant (β = −0.021). Overall, the SEM is consistent with the interpretation that higher inputs lower product-level intensity only when they are effectively converted into biomass and yield. The model is an associational summary of observed variables and does not establish an independent physiological mechanism.

## 4 Discussion

### 4.1 Seasonal consistency of the carbon dilution effect

To further elucidate mechanisms across seasons, we developed season-specific SEMs for the spring and autumn crops and present them together for comparison (**Figure 10**). The growth chain shows that WFI positively drove vegetative biomass (spring β = 0.893; autumn β = 0.800), which promoted biomass accumulation at maturity (spring β = 0.654; autumn β = 1.051), and ultimately increased yield (spring β = 0.394; autumn β = 0.367). WFI had a direct positive effect on yield (spring β = 0.522; autumn β = 0.450). The carbon pool mechanism indicates that the vegetative-stage carbon pool was determined by vegetative biomass, while the maturity-stage carbon pool was influenced by both maturity biomass and the vegetative-stage carbon pool, reflecting a “carry-over” effect of carbon accumulation.

**Figure 10:**
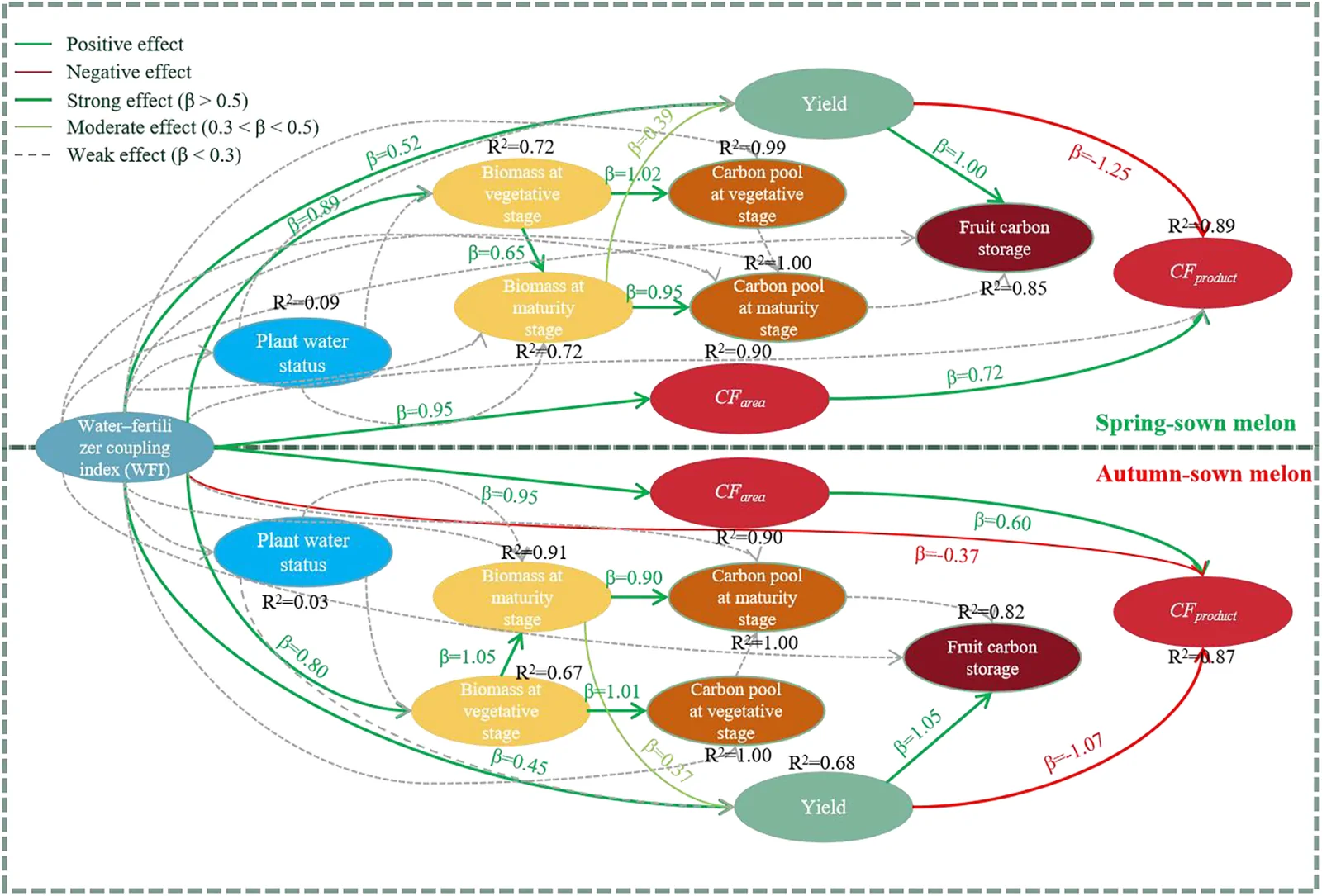
Season-specific structural equation models for spring and autumn crops. Values are standardized path coefficients. Because *CF_product_* is calculated as *CF_area_*/yield, the *CF_area_*→*CF_product_* and yield→*CF_product_* paths are interpreted as model associations rather than independent causal effects.

On the carbon footprint side, a key trade-off was consistently observed: WFI strongly increased *CF_area_* (β = 0.950 in both seasons), indicating that intensified irrigation and fertilization increased emissions per unit area. *CF_product_*, however, was determined by two opposing effects: the emission-increasing effect of *CF_area_* (spring β = 0.729; autumn β = 0.603) and the yield-driven dilution effect (spring β = −1.253; autumn β = −1.067). The direct effect of WFI on *CF_product_* was not significant in either season, confirming that water–fertilizer coupling affects *CF_product_* mainly through its indirect effects on *CF_area_* and yield. These consistent patterns across seasons further support the conclusion that effective conversion of inputs into yield is critical for achieving simultaneous yield gains and reductions in *CF_product_*.

### 4.2 Why does the carbon dilution effect dominate? A physiological hypothesis

In the SEM analysis presented in **Figure 10**, product carbon-footprint intensity was related to two opposing standardized paths: *CF_area_* was positively associated with *CF_product_* (β = 0.729), whereas yield was negatively associated with *CF_product_* (β = −1.253). Within the fitted model, the negative yield-associated coefficient was larger in magnitude. These values are reported with their figure context and are interpreted cautiously because *CF_product_* is calculated from *CF_area_* and yield.

Conceptually, this finding aligns with the notion of “environmental efficiency” highlighted by Poore and Nemecek (2018), who showed that environmental impacts can differ by orders of magnitude among producers and can often be reduced substantially through management optimization. The carbon dilution effect we quantify is essentially a mechanistic realization of that broader principle: high yield is the vehicle through which efficiency gains are expressed in this system.Our results extend this principle to greenhouse fruit production by providing a mechanistic interpretation of how such efficiency gains can be realized: high yield dilutes fixed carbon inputs (plastic film and machinery) and improves the conversion efficiency of variable inputs (fertilizer and electricity). From a broader production-system perspective, this is consistent with the “optimized water–nitrogen mitigation” phenomenon reported in intensively managed wheat–maize systems, where irrigation and nitrogen optimization maintained or increased yield while lowering emission intensity (Zhang et al., 2020). The quantified dominance of the dilution effect (β = −1.253) over the emission-increasing effect (β = 0.729) explains why high-input systems are not necessarily high-carbon systems: the key is whether inputs are effectively converted into yield (Matson et al., 1998). Recent studies similarly demonstrate that coordinated water–fertilizer management and improvements in input-use efficiency can maintain productivity while reducing carbon- and water-footprint intensity (Li et al., 2025; Shi et al., 2026).

We propose that the physiological basis of this conversion lies in the allocation pattern of photosynthetic carbon under different water–fertilizer regimes. Under adequate irrigation (W4), high fertilization (F3) maintains high stomatal conductance and photosynthetic rate throughout the reproductive period. Wang et al. (2018) demonstrated that stomatal aperture, rather than nitrogen nutrition level, is the dominant determinant of water-use efficiency in tomato under fertigation, implying that water supply constrains the realized benefit of fertilizer. In our system, this is supported by the higher biomass accumulation at maturity under W4F3 (70.9 g plant^-^¹ in spring) compared with W1F3 (30.4 g plant^-^¹). Importantly, the additional carbon fixed under W4F3 is not merely stored in vegetative tissues as structural carbohydrates; a substantial proportion is exported to fruits (181.6 g C fruit^-^¹ in spring W4F3), representing productive carbon that directly contributes to yield.

In contrast, under water-limited conditions (W1), the same high fertilization rate fails to sustain stomatal opening, leading to feedback inhibition of photosynthesis (Wang et al., 2018). The carbon that is fixed is disproportionately allocated to osmotic adjustment compounds (e.g., soluble sugars and proline), which increase organ carbon concentration but contribute little to biomass or yield. This explains the “carbon concentration” phenomenon observed in W1F3: high carbon percentage (leaves 52.0%, stems 49.5%) but low total carbon storage (15.18 g C plant^-1^). This stress-induced defensive strategy sacrifices growth and yield and therefore contributes little to the overall carbon sink function, consistent with the general principle that carbon allocation shifts toward defense compounds under stress (Lal, 2004).

The observed pattern is biologically consistent with more effective conversion of water and fertilizer into harvestable yield under adequate irrigation. However, the SEM alone cannot distinguish statistical coupling from physiological causation. The biomass and fruit-carbon observations provide supporting, rather than definitive, physiological evidence for the proposed interpretation.

This physiological perspective has an important implication: efforts to reduce the carbon footprint of agricultural inputs should not focus solely on reducing input levels, but rather on ensuring that inputs are converted into yield. In this system, the yield ceiling is constrained by water availability; once water is adequate, additional fertilizer can be effectively assimilated into productive carbon, amplifying the dilution effect rather than adding to wasteful emissions. This aligns with the “fertilize based on water” principle (Bar-Yosef, 1999; Li et al., 2020) and reinforces the conclusion that water is the foundational resource upon which fertilizer efficiency depends.

### 4.3 Trade-offs between yield, resource efficiency and net carbon benefit

In the autumn crop, W2F2 achieved the highest water use efficiency (18.82 kg m^-3^), but its yield (48.73 t ha^-1^) was still lower than that of W4F3 (52.79 t ha^-1^), revealing a typical trade-off between maximizing yield and maximizing water productivity. Similar trade-offs have been reported in cereal systems, indicating that maximizing a single efficiency metric can come at the expense of other objectives (Zhang et al., 2020). In practice, management should be goal-oriented and account for local water availability. In regions with severe water scarcity, W2F2 may be preferred to maximize water productivity, whereas in areas with relatively sufficient water supply and a strong yield target, W4F3 is more advantageous.

The patterns of PFP further highlight the foundational role of water: under low irrigation, increasing fertilization sharply reduced PFP, whereas under medium-to-high irrigation, higher fertilization maintained comparatively high PFP. This provides a clear field-management warning: fertilization should be determined by water availability (“fertilize based on water”), and blind topdressing under water limitation should be avoided.

### 4.4 Limitations and future research directions

First, the main system boundary excluded measured soil N_2_O emissions and changes in soil organic carbon (Qasim et al., 2021). The supplementary IPCC Tier 1 calculation suggested that direct fertilizer-induced N_2_O would be approximately 0.32–0.40 t CO_2_-eq ha^-1^ across F1–F3. This magnitude does not substitute for field measurement and cannot resolve irrigation effects on soil N_2_O; future studies should monitor treatment-specific fluxes and soil carbon changes before integrating them into a complete footprint.

Second, the recommended management option (W4F3) is based on one year of data and lacks multi-year replication. Interannual climate variability and cultivar differences may affect the robustness of conclusions (Verchot, 2026). Future studies should test multiple growing seasons and cultivars to evaluate generality.

Third, a complete water footprint was not calculated because pollutant leaching loads and the concentration data required for grey-water estimation were unavailable. This limitation is particularly relevant because drip fertigation can substantially influence nitrogen leaching in solar-greenhouse vegetable production systems (Zhang et al., 2019). WUE should therefore be interpreted as an irrigation-productivity indicator rather than a comprehensive water-sustainability metric. SEM identifies multivariate statistical pathways among observed variables but does not dynamically represent soil–plant–atmosphere processes; future work could link process-based crop or soil-water models with LCA when the necessary time-series data become available.

## 5 Conclusions

This study shows that water availability determines whether additional fertilizer is converted into productive biomass and yield or contributes primarily to a higher input-related emission burden. Across both crops, higher yield was consistently associated with lower product carbon-footprint intensity even though area-based emissions increased with input level. Under the tested sandy-soil, cultivar and one-year greenhouse conditions, W4F3 (approximately 3420 m^3^ ha^-1^ irrigation and 863 kg ha^-1^ fertilization) achieved the highest yields (75.74 and 52.79 t ha^-1^) and the most favorable overall balance among yield, plant carbon storage and product intensity. This treatment should therefore be regarded as the best-performing option within the tested range, not as a universally optimal prescription.

## Data Availability

The original contributions presented in the study are included in the article/**Supplementary Material**. Further inquiries can be directed to the corresponding author.
