# Supplementary material for: Water–fertilizer coupling balances yield and the input-related carbon footprint in double-cropping greenhouse Hami melon
Source: Front Plant Sci. 2026 Oct 1;17:1917283. doi: 10.3389/fpls.2026.1917283 (PMC13668531; doi:10.3389/fpls.2026.1917283)
Supplement: Supplementary file 1 [file Table1.docx]

**Table S1. Area-based input-related carbon footprint (CFarea) for all irrigation–fertilization treatments in the spring and autumn crops.**

| Treatment | Spring CFarea (kg CO_2_-eq ha^-1^) | Autumn CFarea (kg CO_2_-eq ha^-1^) |
| --- | --- | --- |
| W1F1 | 8753.95 | 8604.15 |
| W1F2 | 9450.94 | 9301.14 |
| W1F3 | 10147.93 | 9998.13 |
| W2F1 | 8957.50 | 8807.70 |
| W2F2 | 9654.49 | 9504.69 |
| W2F3 | 10351.48 | 10201.68 |
| W3F1 | 9161.05 | 9011.25 |
| W3F2 | 9858.04 | 9708.24 |
| W3F3 | 10555.03 | 10405.23 |
| W4F1 | 9364.60 | 9214.80 |
| W4F2 | 10061.59 | 9911.79 |
| W4F3 | 10758.58 | 10608.78 |
| W5F1 | 9568.15 | 9418.35 |
| W5F2 | 10265.14 | 10115.34 |
| W5F3 | 10962.13 | 10812.33 |

*Note: CF_area_ includes the embodied emissions of fixed inputs, fertilizer inputs and irrigation electricity within the stated input-related system boundary. Direct field N_2_O and CH_4_ fluxes are excluded.*
